# Comparative Molecular Modeling Study of *Arabidopsis* NADPH-Dependent Thioredoxin Reductase and Its Hybrid Protein

**DOI:** 10.1371/journal.pone.0046279

**Published:** 2012-09-27

**Authors:** Yuno Lee, Songmi Kim, Prettina Lazar, Jeong Chan Moon, Swan Hwang, Sundarapandian Thangapandian, Youngsik Shon, Kyun Oh Lee, Sang Yeol Lee, Keun Woo Lee

**Affiliations:** Division of Applied Life Science (BK21 Program), Systems and Synthetic Agrobiotech Center, Plant Molecular Biology and Biotechnology Research Center, Research Institute of Natural Science, Gyeongsang National University, Jinju, Gyeongsangnam-do, Republic of Korea; Semmelweis University, Hungary

## Abstract

2-Cys peroxiredoxins (Prxs) play important roles in the protection of chloroplast proteins from oxidative damage. *Arabidopsis* NADPH-dependent thioredoxin reductase isotype C (AtNTRC) was identified as efficient electron donor for chloroplastic 2-Cys Prx-A. There are three isotypes (A, B, and C) of thioredoxin reductase (TrxR) in Arabidopsis. AtNTRA contains only TrxR domain, but AtNTRC consists of N-terminal TrxR and C-terminal thioredoxin (Trx) domains. AtNTRC has various oligomer structures, and Trx domain is important for chaperone activity. Our previous experimental study has reported that the hybrid protein (AtNTRA-(Trx-D)), which was a fusion of AtNTRA and Trx domain from AtNTRC, has formed variety of structures and shown strong chaperone activity. But, electron transfer mechanism was not detected at all. To find out the reason of this problem with structural basis, we performed two different molecular dynamics (MD) simulations on AtNTRC and AtNTRA-(Trx-D) proteins with same cofactors such as NADPH and flavin adenine dinucleotide (FAD) for 50 ns. Structural difference has found from superimposition of two structures that were taken relatively close to average structure. The main reason that AtNTRA-(Trx-D) cannot transfer the electron from TrxR domain to Trx domain is due to the difference of key catalytic residues in active site. The long distance between TrxR C153 and disulfide bond of Trx C387-C390 has been observed in AtNTRA-(Trx-D) because of following reasons: i) unstable and unfavorable interaction of the linker region, ii) shifted Trx domain, and iii) different or weak interface interaction of Trx domains. This study is one of the good examples for understanding the relationship between structure formation and reaction activity in hybrid protein. In addition, this study would be helpful for further study on the mechanism of electron transfer reaction in NADPH-dependent thioredoxin reductase proteins.

## Introduction

Redox regulation plays an important role in a variety of biological processes. Thus, in order to maintain redox homeostasis, cells have developed many compartmentalized enzymatic and non-enzymatic antioxidative systems. Some of the most well-known antioxidative enzymes belong to the peroxiredoxin (Prx) family, and these abundant proteins are present in a wide range of organisms [1], [2], [3]. Detoxification of the peroxide cofactors of these proteins is carried out through catalytic cysteine residues and thiol-containing reductants [4], [5]. The cysteine residue is oxidized by peroxides during the catalytic cycle, and it is regenerated through intra- or intermolecular disulfide bond formation [6] via numerous reducing systems, such as thioredoxin (Trx), glutaredoxin (Grx), cyclophilin, or AhpF and AhpD [7], [8], [9], [10].

It is already a proven fact that 2-Cys Prxs play important roles in the antioxidative defense systems of plant chloroplasts. A cDNA encoding an NADPH-dependent thioredoxin reductase (NTR) isotype C in *Arabidopsis* was identified and designated AtNTRC. And it was demonstrated that this protein has an effect on efficient transfer of electrons from NADPH to the 2-Cys Prxs of chloroplasts [11], [12]. AtNTRC contained N-terminal thioredoxin reductase (TrxR) and C-terminal Trx domains. It exhibited both TrxR and Trx activities and co-localized with 2-Cys Prx-A in chloroplasts [13]. According to the previous experimental results it was suggested that AtNTRC functions as an electron donor for plastidial 2-Cys Prxs and represents the NADPH-dependent TrxR/Trx system in chloroplasts [14].

**Figure 1:**
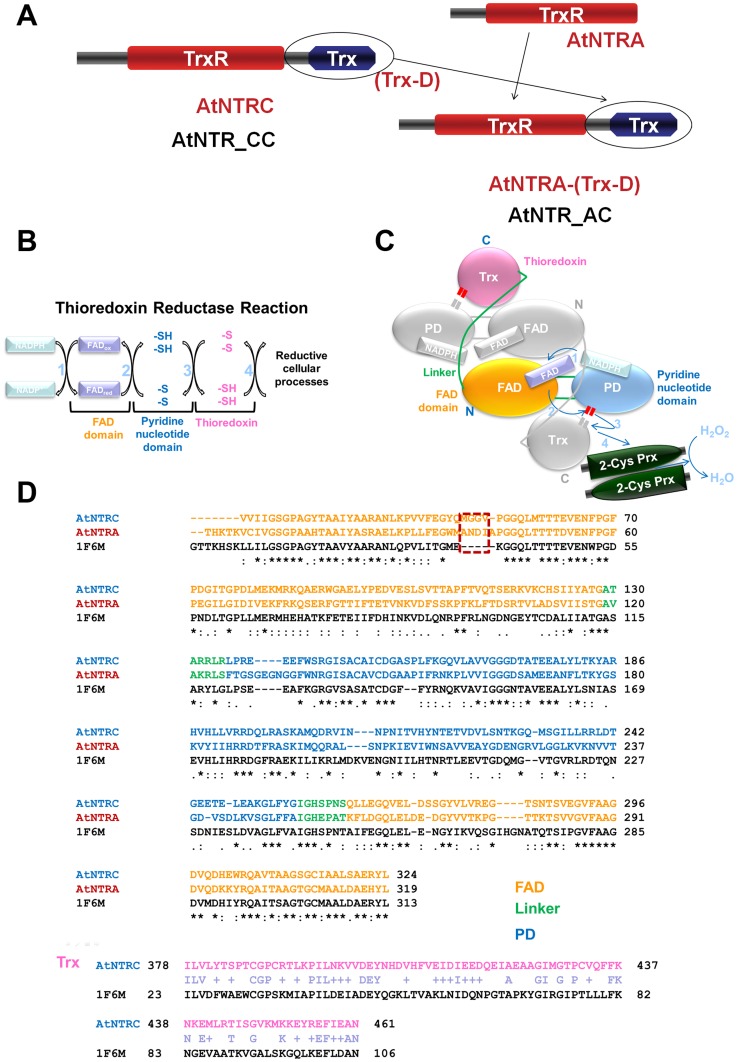
Introduction of proteins and inter-subunit electron transfer reaction with multiple sequence alignment for all proteins. (A) Schematic diagram of protein complex composition with corresponding naming convention AtNTR_CC (AtNTRC) and AtNTR_AC (AtNTRA-(Trx-D)) for the modeling systems. The reaction steps represented by focusing on (B) reducing equivalents and (C) domain structures. Reducing equivalents are transferred from NADPH to a number of enzymes such as peroxiredoxin (Prx) and ribonucleotide reductase through the reactions which are divided into 4 steps. To clearly introduce dimeric structure, the FAD, PD, and Trx domains of one subunit colored in orange, blue, and magenta, respectively and those of anothor subunit in gray. (D) Comparison of TrxR domain between AtNTRC and AtNTRA and sequence alignment for homology modeling with the template *E. coli* TrxR. Different resiudes, which play key role in inducing structural difference of the linker binding between two proteins, are marked by dotted box. Showing multiple sequence alignment of the template *E. coli* TrxR with AtNTR_CC and AtNTR_AC, indicating the functional regions in different colors. Trx part of *E. coli* TrxR was aligned with that of AtNTR_CC system.

In *Escherichia coli* TrxR, cycles of reduction and reoxidation of the flavin adenine dinucleotide (FAD) cofactor depend on rate-limiting rearrangements of the FAD and NADPH domains [15]. The amino acid composition of both AtNTR isotype A (AtNTRA) and AtNTRC sequences comprises important features like FAD domain, pyridine nucleotide domain (PD), the linker region between FAD and Trx, and Trx domain. Reducing equivalents are transferred from NADPH to the flavin cofactor, which is then transferred to the enzyme disulfide (Cys150–Cys153), leading to the disulfide formation (Cys387–Cys390) of the oxidized Trx domain (Figure 1B) [16]. An inter-subunit pathway of electron transfer during this catalysis has been proposed by Perez-Ruiz & Cejudo [17] suggesting an electron transfer from the PD domain of one subunit to the Trx domain of the other subunit (Figure 1C). Reduced Trx domain is a reductant for chloroplastic 2-Cys Prx-A as the final electron acceptor. AtNTRA, contains only TrxR domain, but AtNTRC consists of N-terminal TrxR and C-terminal Trx domains. Hence, a hybrid fusion of AtNTRA sequence with the C-terminal Trx domain sequence from AtNTRC was constructed (Figure 1A). This hybrid protein is named hereafter as AtNTRA-(Trx-D) and used to confirm the importance of Trx domain in AtNTRA.

AtNTRC has various oligomer structures [18], [19], especially active form of NTRC is the dimer, and Trx domain is important for chaperone activity. Our previous experimental study has reported that the hybrid protein has formed variety of structures and shown strong chaperone activity. But, electron transfer mechanism was not detected at all [14]. We have expected that AtNTRC and AtNTRA-(Trx-D) will be same in terms of their structures and show similar activity but it showed dramatically decreased electron transfer. This kind of electron transfer problem was observed in reaction 3 of the TrxR catalysis process (Figure 1B and 1C). The initial homology models for the two proteins were generated using *E. coli* TrxR-Trx complex (PDB ID: 1F6M) as template, which is the flavin-reducing (FR) conformation. In order to elucidate the atomic structural details behind this behavior of AtNTRA-(Trx-D), we performed two different molecular dynamics (MD) simulations of AtNTRC and AtNTRA-(Trx-D) proteins with same cofactors such as NADPH and flavin adenine dinucleotide (FAD). Structural differences were found from superimposition of two representative structures which were the closest conformations to average structure for last 10 ns. The main reason for the inability of AtNTRA-(Trx-D) to transfer electron from TrxR domain to Trx domain was found out by analyzing the differences in the active site key catalytic residues. Our study reveals that the differential behavior of AtNTRA-(Trx-D) may have occurred due to not only structural differences in the linker region but also dissimilar interface interactions of Trx domain.

## Results

### Sequence comparison of enzymes AtNTRC & AtNTRA-(Trx-D)

AtNTRA was connected with Trx domain of AtNTRC to confirm the role of Trx domain with AtNTRA. The AtNTRC sequence consists of 443 residues excluding the first 18 residues. Figure 1A explains clearly the target sequence preparation for the enzymes to be modeled. For our study, the molecular modeling strategy and the naming conventions were adopted based on their sequence relationship. The AtNTR_CC and AtNTR_AC were defined as names for the modeled systems AtNTRC and AtNTRA-(Trx-D), respectively (Figure 1A). Comparison of amino acid composition of both the enzyme sequences showed 49.4% identity and 73% similarity. Functional domains like FAD, the linker region between FAD and Trx, PD and Trx domain were well aligned in both the enzymes and along with the important cysteine residues (Figure 1D). The sequence alignments of the template with AtNTR_CC and AtNTR_AC have shown the identity of 48% and 45% along with 66% and 64% sequence similarity, respectively (Figure 1D). This indicates that both the enzymes must have similar functional characteristics.

### Construction of reasonable dimer structures for AtNTR_CC and AtNTR_AC

**Figure 2:**
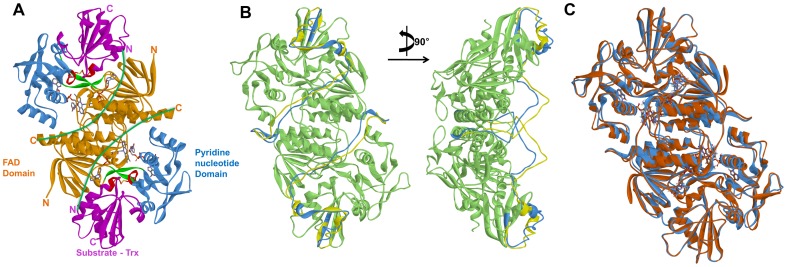
Three dimensional structures of template and the two proteins with refined linker region by 20 ns MD simulation. (A) The template *E. coli* TrxR-Trx complex representing FAD (orange), PD (blue), and Trx (magenta) domains and functional regions (red) along with the missing linker region (green). (B) Showing the modeled missing linker region for the AtNTR_CC before (yellow) and after (light blue) 20 ns MD simulation. (C) Showing the superimposed homology modeled structures of AtNTR_CC (light blue) and AtNTR_AC (light brown), indicating the functional domains in different colors along with cofactors.

The dimeric structures were generated using dimeric structure of *E. coli* TrxR-Trx complex (PDB ID: 1F6M) as template (Figure 2A), which is the flavin-reducing (FR) conformation, for the systems AtNTR_CC and AtNTR_AC. The AtNTR_CC homology model was built by omitting the first 18 residues at the N-terminal region, since no sequence similarity was found for N-terminal residues during the pairwise sequence alignment. The missing linker region was treated as flexible loop which is connecting C-terminus in FAD domain of one subunit to the N-terminus in Trx domain of the other subunit. Subsequently, the linker region was refined by 20 ns MD simulation with restraining the other parts. Although the NTRC monomer model using *Arabidopsis thaliana* NTR and *Escherichia coli* Trx as templates was previously suggested [20], this dimeric structure of the AtNTRC including refined linker region is firstly introduced in this study.

The well refined homology models were thus obtained for both the enzymes with evaluation of their structure quality showing 81.3% residues in most favored region and Z-score: −8.69 for AtNTR_CC system and 86.1% residues and −9.69 for AtNTR_AC system unlike the template protein which has 80.9% of residues (Figure S1). The complexes were constructed along with the oxidized form of FAD & NADP^+^ cofactors. The superimposed structural representation of the two enzymes clearly defined the position of the cofactors and the functional domains. The structural root-mean-square deviation (RMSD) of the modeled protein structure of these two enzymes is 0.15 nm, which indicates that they have similar secondary structure elements, having only differential variable loop arrangements (Figure 2C).

### Representative structure and stabilities of two systems

**Figure 3:**
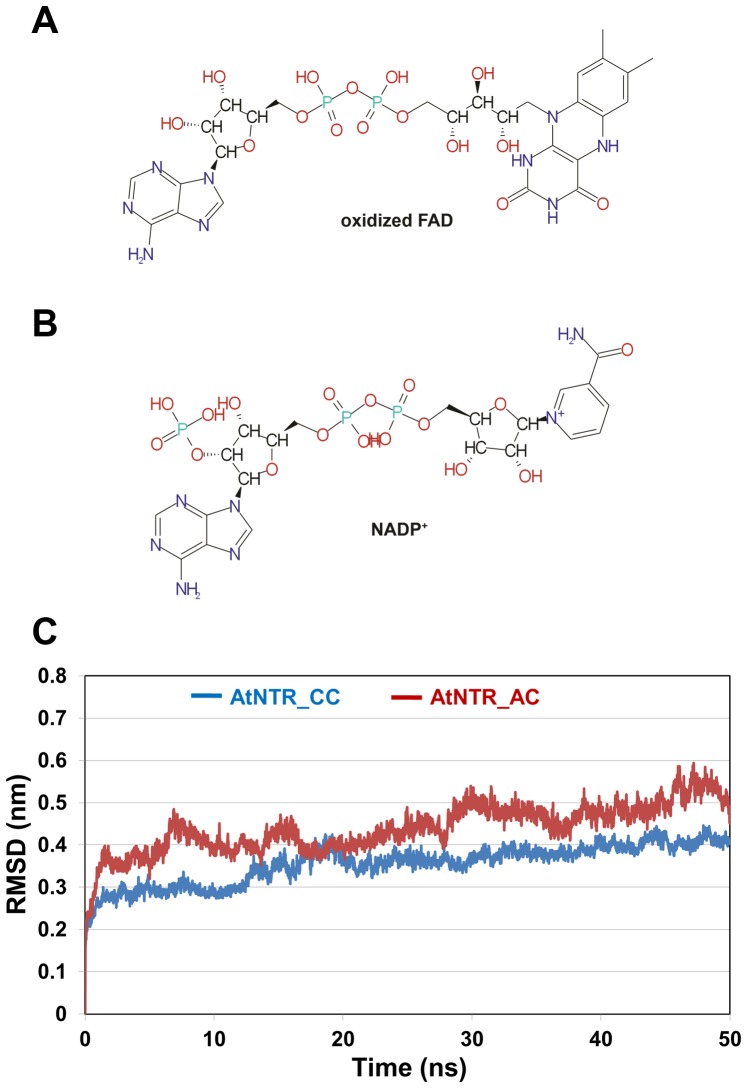
Preparation of cofactors for MD simulation and result of RMSD analysis. Chemical structures of (A) oxidized FAD and (B) NADP^+^ are used in MD simulations. (C) Cα RMSDs are plotted from the initial structure in contrast with time for the AtNTR_CC (blue) and AtNTR_AC (red) MD simulations.

The final modeled protein structures were used as initial structure with oxidized FAD and NADP^+^ obtained from the template in complex with cofactors (Figure 3A and 3B). Main objectives of MD simulations performed in this study are firstly to find out the proper adjusted structure of AtNTR_CC which is different from the template and then secondly to identify the reason why AtNTR_AC cannot transfer the electron from TrxR domain to Trx domain and to verify the conformational changes taking place in both AtNTR_CC and AtNTR_AC systems in presence of the cofactors like oxidized FAD & NADP^+^ and Trx. The details of the MD simulation environments and the size of two systems are listed in Table 1. In order to compare the protein structures from the two different systems, their representative structures of AtNTR_CC (45,780 ps) and AtNTR_AC (45,290 ps) were selected from each simulation (last 10 ns) and those are the closest conformation to the average structure.

**Table 1:** Summary of model systems for MD simulations.

Protein	Acronym	No. of Atoms	Time	Status
AtNTRC	-	135,432	20 ns	Refinement of the linker region with restraining the other parts
AtNTRC	AtNTR_CC	141,972	50 ns	Natural form
AtNTRA-(Trx-D)	AtNTR_AC	142,114	50 ns	Hybrid form
AtNTRA and Trx domain complex	-	142,143	10 ns	AtNTR_AC without the linker region

**Figure 4:**
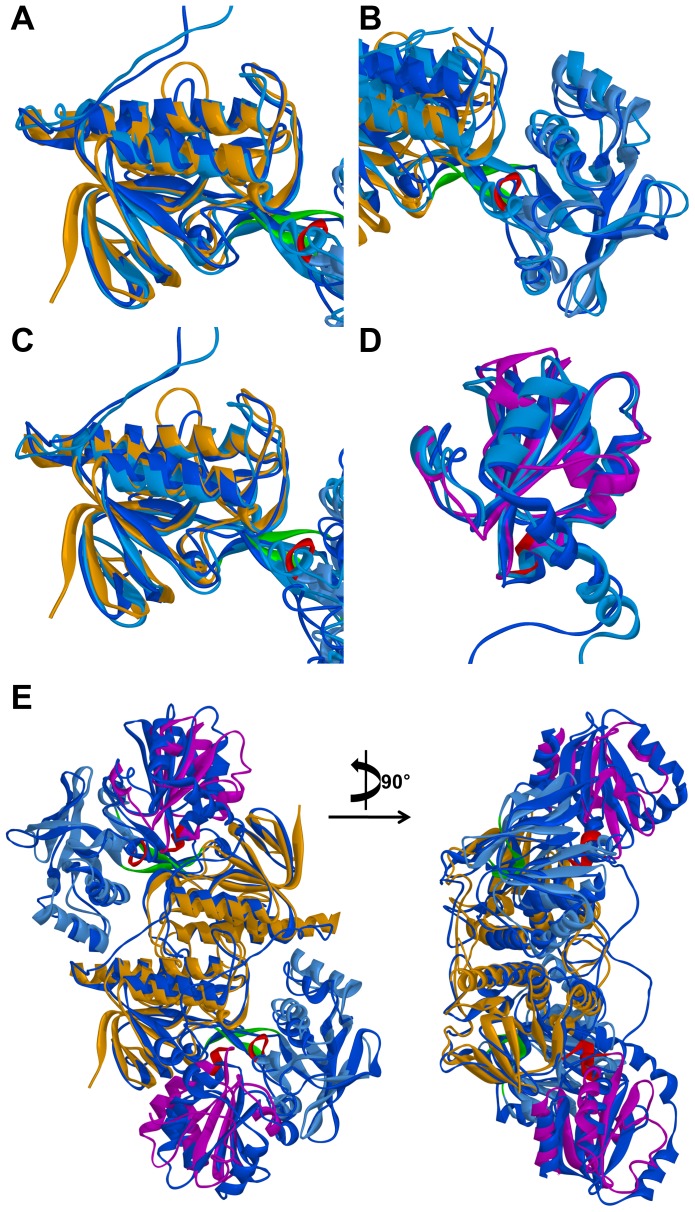
Comparison of AtNTR_CC representative structure and template *E. coli* TrxR-Trx complex structure by superimposition of each domain. (A) FAD upper region, (B) PD, (C) FAD lower region, (D) Trx, and (E) all NTR regions.

The calculated average Cα RMSD of each system during the last 10 ns is 0.41 and 0.51 nm, respectively (Figure 3C). Whereas the RMSD value of AtNTR_CC system is converged after 20 ns and adjusted stably, but AtNTR_AC system is more unstable than the AtNTR_CC. In order to check the structural difference between AtNTR_CC and *E. coli* TrxR-Trx complex, the representative structure of AtNTR_CC was superimposed with crystal structure of the complex by each domain (FAD upper region, PD, FAD lower region, Trx, and NTR region). Each obtained RMSD from superimposition for subunit A is 0.15, 0.20, 0.21, 0.20, and 0.26 nm, and the RSMD for subunit B is 0.17, 0.25, 0.15, 0.17, and 0.26 nm, respectively (Figure 4). For comparing the relative position of Trx domain, both the dimeric structures were superimposed by whole NTR regions (Figure 4E). The RMSD obtained from superimposition is 0.27 nm indicating that the both AtNTR_CC and *E. coli* TrxR-Trx complex structures are relatively similar with each other. From these results, we can suggest that the adjusted structure of AtNTR_CC with well maintaining the orientation of the crystal structure was obtained from the 50 ns MD simulation.

### Structural difference of two systems

**Figure 5:**
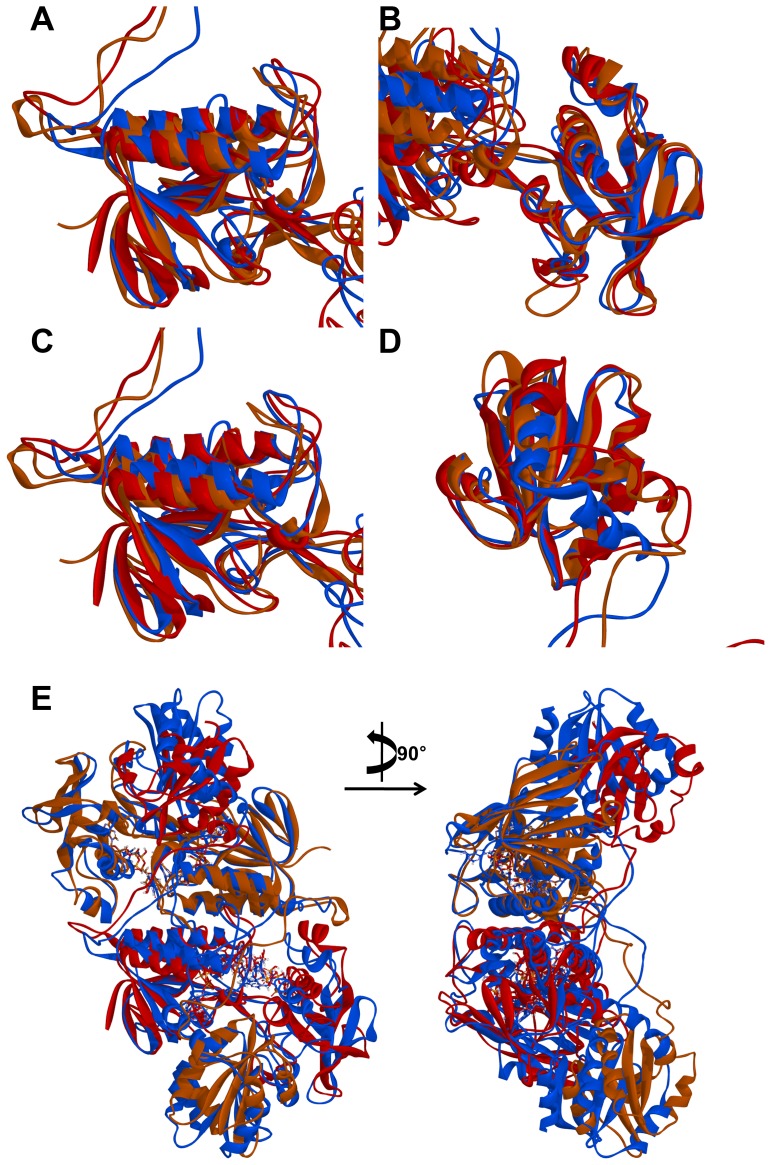
Specific domain-wise superimpositions of the two representative structures. Superimposed structures based on (A) FAD upper region, (B) PD, (C) FAD lower region, (D) Trx, and (E) all NTR regions.

Structural differences were found from superimposition of two representative structures. An RMSD of 0.57 nm was observed between the two entire structures of AtNTR_CC and AtNTR_AC systems. Specific domain-wise (FAD upper region, PD, FAD lower region, the linker region, Trx, and NTR region) superimpositions of the representative structures were measured and the respective average RMSD values (0.17, 0.29, 0.25, 0.56, 0.20, and 0.34 nm) of all comparisons were monitored (Figure 5 and Table 2). The most structural difference was observed in the linker and NTR regions with 0.56 and 0.34 nm RMSD, respectively. Hence, the significant conformational changes were noticed to be influenced by the linker and NTR regions emphasizing the importance of these domains in the enzyme, both structurally and functionally. To identify the relative position of Trx domain between two systems, both the dimeric structures were superimposed based on all NTR regions. The Trx domains of AtNTR_AC was significantly shifted and bent inside than that of the AtNTR_CC (Figure 5E).

**Table 2:** Specific domain-wise superimpositions of representative structures for AtNTR_CC and AtNTR_AC subunits.

Domain name	AtNTR_CC subunit A		AtNTR_CC subunit B		
	RMSD(nm) AtNTR_AC subunit A	RMSD(nm) AtNTR_AC subunit B	RMSD(nm) AtNTR_AC subunit A	RMSD(nm) AtNTR_AC subunit B	Average
FAD upper region	0.18858	0.14478	0.18912	0.14990	0.168095
PD	0.30917	0.28113	0.30554	0.26828	0.29103
FAD lower region	0.28758	0.27163	0.25641	0.19249	0.252028
The linker region	0.48554	0.49334	0.66165	0.60617	0.561675
Trx	0.18401	0.20260	0.18348	0.24763	0.20443
NTR (FAD+PD)	0.40628	0.39075	0.32255	0.24760	0.341795

**Figure 6:**
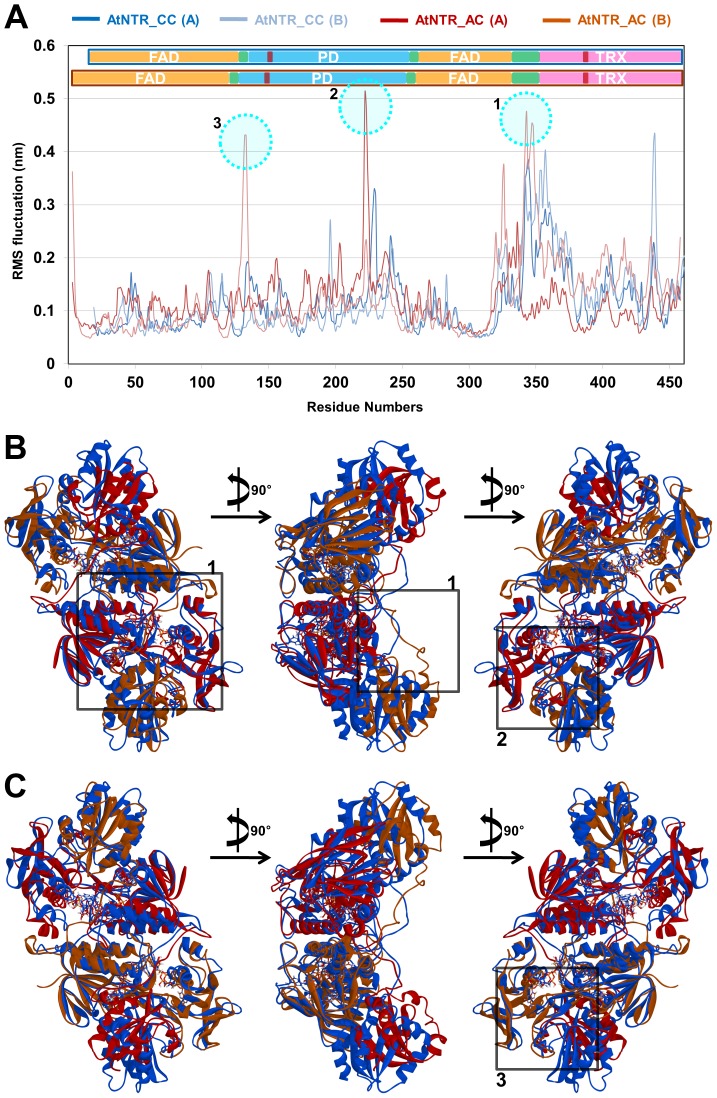
RMSF and entire structure differences with the most fluctuated regions. (A) The most flexible regions such as F342-K350 (of the linker region in subunit B), D221-R225 (of PD in subunit A), and G130–G134 (of PD in subunit B) in AtNTR_AC are emphasized by dotted circle in the RMSF plot. (B) Superimposed structures of two proteins by NTR regions. To clearly show the structural difference of the proteins, all subunits in AtNTR_CC are colored by blue and subunit A and B in AtNTR_AC is colored by red and light brown, respectively. The most flexible regions are highlighted by square block with corresponding numbers. (C) Vertically inverted view of figure 5E with numbered square block indicating the third flexible domain region.

**Figure 7:**
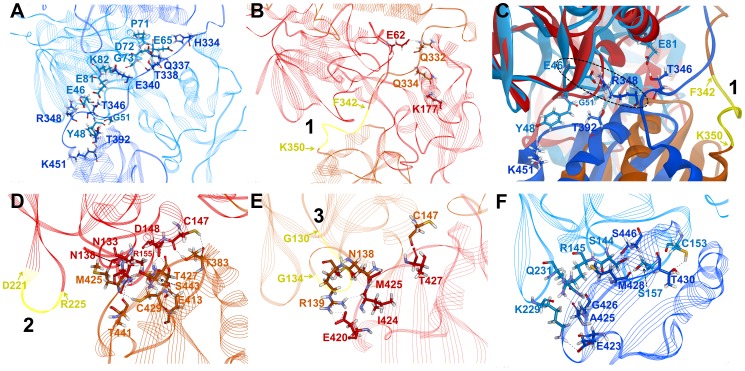
The linker binding and interface interactions with the most fluctuated regions. (A) Stable interactions of the linker region in AtNTR_CC system. Strong hydrogen bonding and hydrophobic interactions were maintained in the interface region between the linker (H334, Q337, T338, E340, T346, and R348) and FAD domain (E46, E65, P71, D72, G73, E81, and K82) of AtNTR_CC system. (B) Unfavorable linker binding of AtNTR_AC system. Unfavorable hydrogen bonding interaction with PD domain is found in AtNTR_AC system. (C) Superimposition of two representative structures showing the difference of the linker binding. Key interaction for the linker binding is highlighted by dotted circle. The flexible region F342-K350 located in the linker region is also emphasized by yellow. (D) Interface interactions of PD (red, subunit A) and Trx (light brown, subunit B) domains with flexible region D221-R225 (yellow). (E) Interface interactions of PD (light brown, subunit B) and Trx (red, subunit A) domains with flexible region G130–G134 (yellow). (F) Interface interactions of PD (light blue, subunit B) and Trx (blue, subunit A) domains in AtNTR_CC. Strong hydrogen bonding and hydrophobic interactions were retained in the interface region between PD (S144, R145, C153, S157, K229, and Q231) and Trx domain (E423, A425, G426, M428, T430, and S446) with showing the important interaction between C153 and T430 for maintaining the distance between catalytic residues.

The average root-mean-square fluctuation (RMSF) of the proteins was measured to examine the overall flexibility during the last 20 ns. Comparative RMSF analysis of AtNTR_CC with AtNTR_AC showed that the linker and PD regions of AtNTR_AC are more flexible than those of AtNTR_CC (Figure 6A). The most flexible regions such as F342-K350 (of the linker region in subunit B), D221-R225 (of PD in subunit A), and G130–G134 (of PD in subunit B) in AtNTR_AC are sequentially numbered and highlighted by dotted circles in the RMSF plot. From the RMSF graph, we found that the PD and the linker regions are the most fluctuated regions in the AtNTR_AC. To clearly show the difference of flexible region between two systems, NTR region of subunit B in AtNTR_CC was located at bottom side in Figure 6B and 6C and NTR regions of subunits B and A in AtNTR_CC were superimposed with those of subunits A and B in AtNTR_AC. Figure 6C is the vertically inverted view of figure 5E with numbered square block indicating the third flexible domain region. Detailed view of the most flexible regions is shown in figure 7 for providing information of the different interacting residues between two systems.

### Interactions between the linker and FAD/PD domain

The differences in the interaction of the linker region between two systems are depicted in figure 7A and 7B. The AtNTR_CC system retains the stable interaction between the linker and FAD domain while the AtNTR_AC system rarely interacts with the FAD domain. Interestingly, the interaction between the linker and the PD domain was observed in AtNTR_AC. It seems that the linker should not interrupt the rotation of PD domain for progress to the next reaction. We think that this unfavorable interaction can cause the problem for electron transfer function of the hybrid protein. Strong hydrogen bonding and hydrophobic interactions were found in the interface region between the linker (H334, Q337, T338, E340, T346, and R348) and FAD domain (E46, E65, P71, D72, G73, E81, and K82) of AtNTR_CC system (Figure 7A). However, in the AtNTR_AC system, the most hydrogen bonds between corresponding residues were not detected (Figure 7B). In detail, some flexible residues in the linker region highlighted by yellow, first one of the three flexible regions, have no interactions with other regions in AtNTR_AC system (Figure 7B and 7C). But, in AtNTR_CC system, R348, one of the corresponding residues (F345-K353) has formed strong hydrogen bond with the E46 in FAD domain. These results are the most distinct differences of the interactions of the linker between the two systems.

### Inter-subunit interactions between PD domain of one subunit and Trx domain of the other subunit

The inter-subunit interactions of the shifted Trx domain of the AtNTR_AC were investigated (Figure 7D and 7E). Although many hydrogen bonding interactions were observed in the interface region between the PD domain in subunit A and the Trx domain in subunit B, the shifted Trx domain does not form any interaction with the second flexible region, D221-R225 (Figure 7D). However, in AtNTR_CC, the corresponding residues are involved in hydrogen bond interactions (Figure 7F). In addition, the shifted Trx domain also forms the strong hydrogen bond interaction between T383 and C147 which is one of key residues for the reaction. But, in AtNTR_CC, the corresponding catalytic residue C153 is interacted with T430 rather than the corresponding residue T386. For the interactions between the PD domain in subunit B and the Trx domain in subunit A, this important interaction between C147 and T427 was barely maintained (Figure 7E). Although this inter-subunit interaction includes the key residues, the interactions of the rest parts, especially the third flexible region (G130–G134) located in PD domain, are relatively weak. These results indicate that the shifted Trx domain in AtNTR_AC forms a different or weak interactions comparing to that in AtNTR_CC (Figure 7D and 7E). Hence, the different interaction in AtNTR_AC system can be the one of the reasons for inability of electron transfer reaction of the AtNTR_AC.

### Essential dynamics (ED) analysis

**Figure 8:**
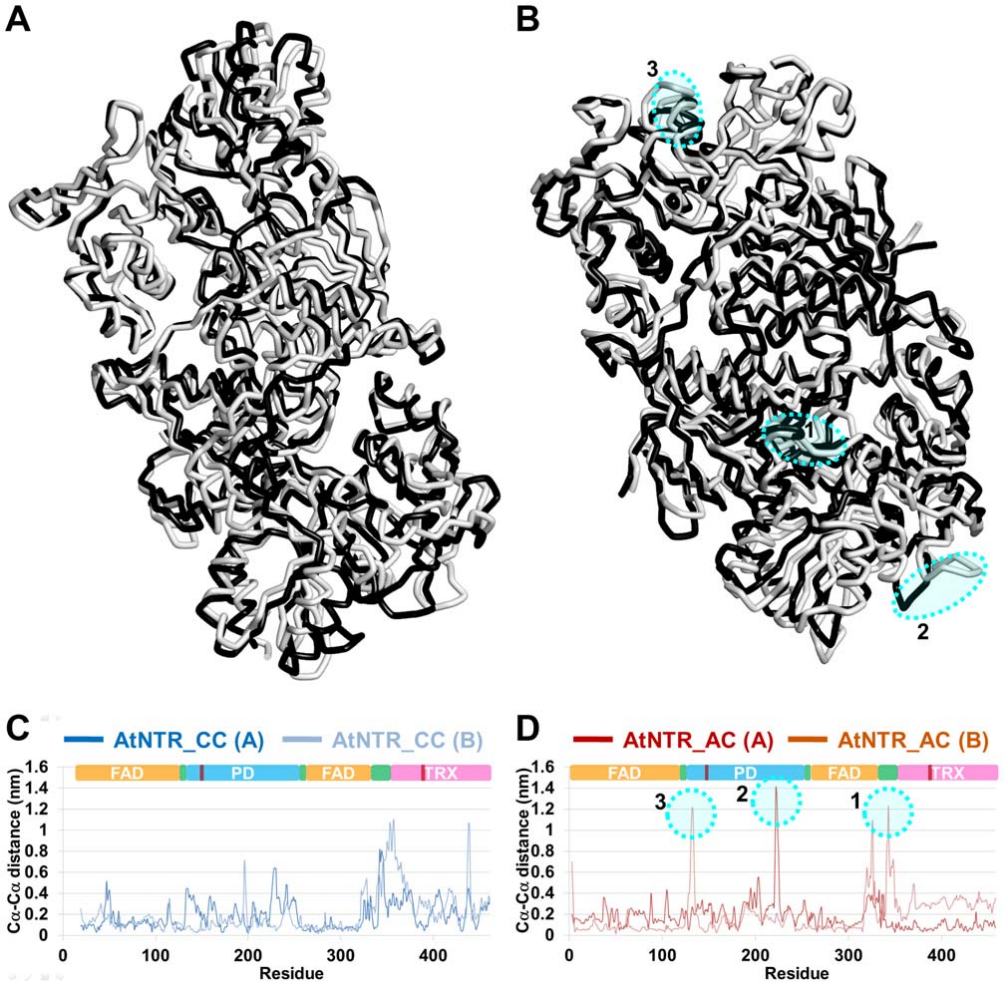
Essential dynamics analysis for the two systems. The two trace structures (minimum in white color and maximum in black color) from the maximal and minimal projections along the largest eigenvector are superimposed in each system, (A) AtNTR_CC and (B) AtNTR_AC. The largest correlated motion for the linker region is emphasized by dotted circle. The *C_α_*-*C_α_* distance plots between the superimposed structures of (C) AtNTR_CC system and (D) AtNTR_AC system are also given to provide quantitative difference values.

The ED analyses were performed to investigate the essential structural movements and to support the two different simulations. We have observed the most dynamic structural change of PD and the linker region in AtNTR_AC system. The qualitative structural analyses show the superimposition of two extreme structures of each system (Figure 8A and 8B). The regions of the most essential motion are highlighted by dotted circle. For a comprehensive view, quantitative plots of *C_α_*-*C_α_* distance between the superimposed two extreme structures were also plotted, which indicated much dynamic motion around the PD and the linker regions of AtNTR_AC system (Figure 8C and 8D). This result is similar with that of the RMSF analysis. Based on the results from the both RMSF and ED analyses for the AtNTR_AC system, we can conclude that the PD and the linker regions are the most fluctuated region and also moved in wide range.

### Binding mode of cofactor conformations

**Figure 9:**
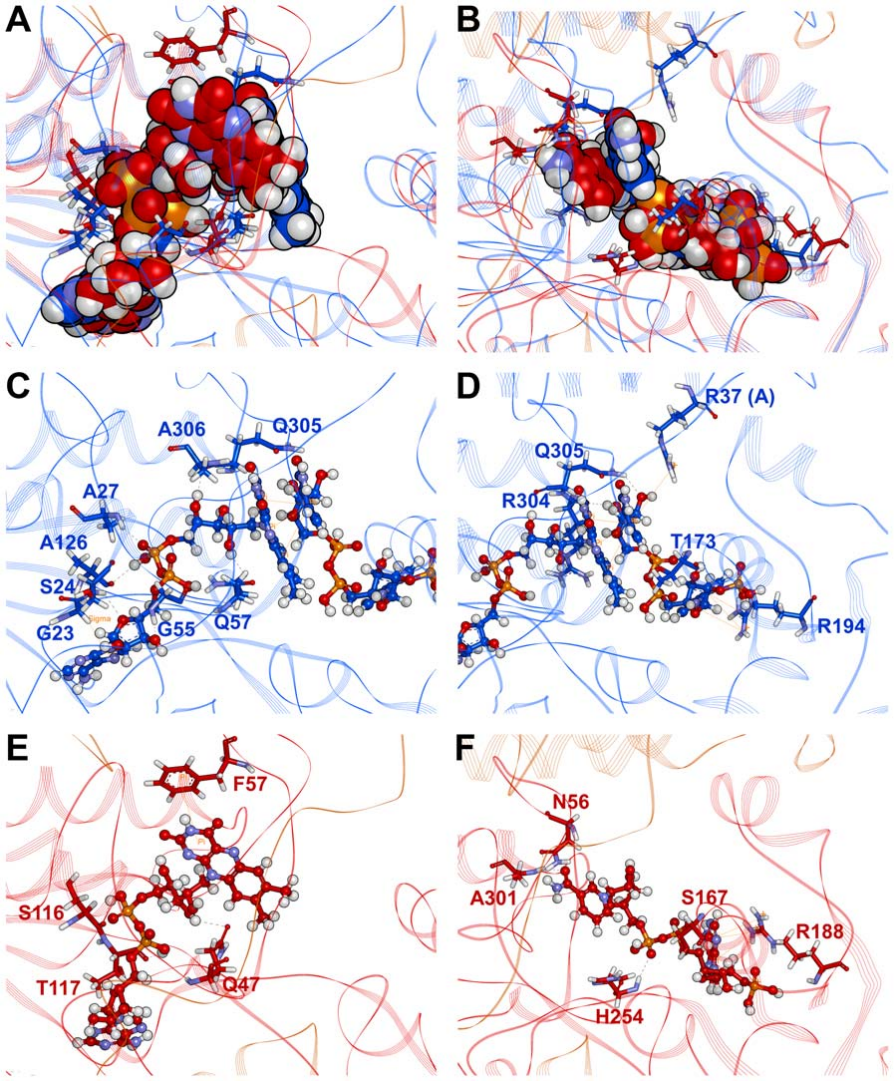
Binding modes of cofactors in the two representative structures. Superimposed structures of (A) oxidized FAD and (B) NADP^+^ in both AtNTR_CC (blue) and AtNTR_AC (red) systems. Binding conformations of (C) oxidized FAD and (D) NADP^+^ in AtNTR_CC system. Binding modes of (E) oxidized FAD and (F) NADP^+^ in AtNTR_AC system. The cofactors are shown in sphere models and hydrogen bonding interactions of the cofactors are represented by dotted lines in black color and pi interactions by orange line.

The binding modes of cofactors (oxidized FAD and NADP^+^) in energy minimized structure were found to be similar in both systems. During the simulation the binding modes of the cofactors were much modified by the conformational changes in the AtNTR_AC system than AtNTR_CC system. Structural analyses were performed to investigate the hydrophobic and hydrogen bonding interaction between cofactors and protein. The results from the protein-ligand interaction analysis are exposed for the comparison of hydrophobic and hydrogen bond interacting residues between the two systems (Table 3). The representative superimposed structures (NTR domain as reference) of both systems revealed the differential binding mode of FAD and NADP^+^ (Figure 9A and 9B). Different hydrogen bonding and hydrophobic interactions were noticed in each system with FAD and NADP^+^. Different pi and hydrogen bond interacting residues were identified for binding of FAD with each system, which include G23, S24, A27, G55, Q57, A126, Q305, and A306 residues in AtNTR_CC system (Figure 9C) and Q47, F57, S116, and T117 residues in AtNTR_AC system (Figure 9E). From figure 9C and 9E, we were able to notice different interaction of cofactor FAD in two systems, especially strong pi-pi interaction with another cofactor NADP^+^ in AtNTR_CC system, which renders more stability to AtNTR_CC than AtNTR_AC system.

**Table 3:** Protein-ligand interactions of two cofactors with AtNTR_CC and AtNTR_AC.

Protein	Ligand	Protein-ligand interactions		
		Hydrogen bonds	Hydrophobic contacts	Pi interaction
**AtNTR_CC (subunit B)**	OxFAD	S24, A27, G55, Q57, A126, A306, Q305	I22, F45, P54, T60, V64, E97, A129, T130, T173, H259, L265, G296	G23 (sigma), NADP^+^ (pi)
	NADP^+^	T173, R304, Q305	V64, V192, L197, G258	R37 (subunit A; cation), R194 (cation), OxFAD (pi)
**AtNTR_AC (subunit A)**	OxFAD	Q47, S116, T117	V11, G12, G36, L48, T52, V54, F60, I69, F73, V89, V120, H254	F57 (pi)
	NADP^+^	N56, S167, H254, A301	-	R188 (cation)

The different interacting residues for the binding of NADP^+^ with each system were R37 (subunit A), T173, R194, R304, and Q305 residues in AtNTR_CC system (Figure 9D) and N56, S167, R188, H254, and A301 residues in AtNTR_AC system (Figure 9F), respectively. From these results, we can clearly see this kind of cofactor binding difference between two systems.

### Structural difference of active site in AtNTR_CC and AtNTR_AC

**Figure 10:**
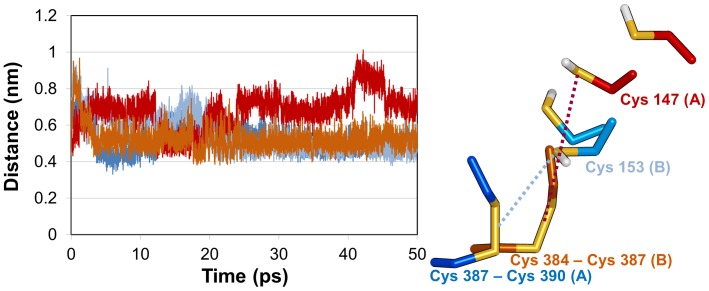
Distance between key cysteine residues. Distance graph (in left) of catalytic sulfide atoms for electron transfer in AtNTR_CC (blue) between Cys153:S (in subunit B) and Cys387–Cys390:S-S (in subunit A) and for that in AtNTR_AC (red) between Cys147:S (in subunit A) and Cys384–Cys387:S-S (in subunit B). In the right panel, the key catalytic residues are represented by stick model.

In order to elucidate the difference of active site, we have calculated the distance between catalytic cysteine residues, which are required for the electron transfer in AtNTR_CC and AtNTR_AC systems (Figure 10). The analysis clearly showed that the distance between the catalytic residues in the AtNTR_CC system continue to be within 0.4–0.5 nm, which is a favorable distance for electron transfer, whereas the distance between the catalytic residues in AtNTR_AC system ranged between 0.7–0.8 nm during last 5 ns. The average distance values for AtNTR_CC and AtNTR_AC systems are 0.46 and 0.72 nm, respectively. Based on the MD analysis and the corresponding results we conclude that the main reason for no electron transfer from TrxR doman to Trx domain in AtNTR_AC is the distorted catalytic cysteine residues. The distance between the catalytic residues of AtNTR_AC is not enough to transfer the electron compared to that of AtNTR_CC.

### Additional MD simulation of AtNTR_AC without the linker region

In order to verify whether the linker region is one of the key factors for inability of electron transfer reaction, additional MD simulation of AtNTR_AC without the linker region was performed during 10 ns. Two snapshot structures which are the closest conformation to the average structure during the last 2 ns were selected as the representative structure for with the linker (9,300 ps) and without the linker (9,050 ps). When the two structures were superimposed by NTR region, relative position difference of Trx domains was observed (Figure S2). Whereas in AtNTR_AC without the linker, the position of the Trx domain was almost same with that in the AtNTR_CC, the domain in the AtNTR_AC with the linker was distinctively shifted and bent inside within 10 ns itself. This result suggests that the unstable linker region in AtNTR_AC has influenced on conformational change of the Trx domain. In overall, these extra results from the additional simulations help us to understand that the linker is very important for the conformation and activity control of this protein.

## Discussion

### Construction of AtNTRC and AtNTRA-(Trx-D) molecular models

The genome of *A. thaliana* contains more than 40 genes encoding either Trx or Trx-like proteins [21]. This outstanding diversity indicates a high degree of specialization and/or a functional redundancy for thiol reductases in plants, which participate in essential processes such as the regulation of photosynthetic activity [22]. In this present study of ours, reasonable molecular models of the enzymes AtNTRC & AtNTRA-(Trx-D) were constructed by homology modeling approach to validate the importance of Trx domain in function. The homology models of AtNTRC and AtNTRA-(Trx-D) were constructed based on the X-ray crystal structures of *E.coli* TrxR-Trx complex. Template of the FR conformation of *E. coli* TrxR-Trx complex was used for building the Trx and TrxR complex structure for the models. Cycles of reduction and reoxidation of the FAD cofactor depend on rate-limiting rearrangements of the FAD and NADPH domains. These two domain orientations permit reduction of FAD and NADPH and oxidation of the enzyme dithiol by Trx [15]. The relative positions of the domains in *A. thaliana* TrxR differ from those of the *E. coli* reductase. Even though crystal structure of TrxR structure from *A. thaliana* is deposited already in PDB [23], we were not able to use it for the homology modeling, since the available structures were flavin-oxidizing (FO) conformation, but not FR.

Our main aim was to target reaction 3 which includes FR and not FO (Figure 1B and 1C). Hence the template structure of TrxR from *E.coli* with an identity of 48% and 45% along with 66% and 64% sequence similarity for AtNTRC and AtNTRA-(Trx-D), respectively, was further used for modeling. The template structure of *E.coli* TrxR-Trx complex has cross-linked active site serines which were mutated from cysteine to serine, so as to be a representative structure of the intermediate state between reaction 3 and 4. For our purpose we have modified this complex structure by mutating serine residue back to cysteine and breaking the cross-link between C32 of Trx and C138 of TrxR and we also created a new disulfide bond between C32 and C35 of Trx domain. Such modeling strategy was employed to obtain the probable structural conformation for the reaction 3.

### Stability and structural difference of AtNTR_CC and AtNTR_AC systems

Our homology models of AtNTRC and AtNTRA-(Trx-D) were structurally similar, inferring that they both should have same functional mechanism. But experimentally both the modeled systems were proven to have different electron transfer mechanism. Our modeling study provides the probable reason behind this difference in function between two structurally similar AtNTRC and AtNTRA-(Trx-D) proteins. Two 50 ns MD simulations (AtNTR_CC and AtNTR_AC) on the proteins with same cofactors such as NADPH and FAD were performed and analyzed to find out the reason of decreased electron transfer from TrxR to Trx domain in the hybrid protein AtNTR_AC.

The Cα RMSD plot showed that the RMSD value of AtNTR_CC system is maintained stably, but AtNTR_AC system is more unstable than the AtNTR_CC. The RMSD value of AtNTR_AC is irregularly increased because of the unstable linker region. However, the conformation of the AtNTR_CC system is well conserved and maintained after adjusting their protein sequence. Structural differences of entire protein and active site have been found from the superimposed representative structures of the two systems. Members of the thioredoxin superfamily are characterized by the presence of a conserved redox-active site, where two cysteine residues are separated from each other by two amino acids [21], [24]. The cysteine residues form a disulfide bridge upon catalysis. For a new catalytic cycle, this disulfide bridge has to be reduced with the participation of thioredoxins (Trxs), of which different types exist in the chloroplast [25]. Based on our MD study on these two modeled systems, the distance between key cysteine residues in active site of AtNTR_CC system is much closer than that of AtNTR_AC system during the simulation time. The flexible and unfavorable linker binding and the subsequent conformational changes in AtNTR_AC have led to the observed differences compared with AtNTR_CC.

### Unstable linker region and shifted Trx domain

In this study, three the most flexible regions were found out in AtNTR_AC such as F342-K350 of the linker region in subunit B, D221-R225 of PD in subunit A, and G130–G134 of PD in subunit B. One of the most flexible regions and the most important region for elucidating the reason of inability of electrons transfer reaction in AtNTR_AC is the linker region. The difference of the linker interactions was clearly observed in superimposition of two representative structures (Figure 7C). From the sequence alignment and structural analyses, we can suggest that this structural difference came from difference of amino acid composition in NTR region between AtNTR_CC and AtNTR_AC, especially Y48-V53 residues (YQMGGV) in AtNTR_CC and W37-I42 residues (WMANDI) in AtNTR_AC (Figure 1D). This Y48-V53 loop in AtNTR_CC has formed a proper exposition of E46 to have strong hydrogen bond interaction with R348 (Figure 7C). In case of AtNTR_AC, there is less of a chance to form this strong interaction because the corresponding residue E35 is formed a hydrogen bond interaction with R74 which is located in FAD upper region.

Due to the unstable linker region in AtNTR_AC, the relative position of Trx domains was more shifted and bent inside than that of the AtNTR_CC (Figure 5E). This is proved by additional MD simulation of AtNTR_AC without the linker region showing the similar orientation with AtNTR_CC (Figure S2). When the linker region is inserted in between NTR and Trx domains, unstable linker region makes conformational changes on Trx domain. The shifted Trx domains in AtNTR_AC have different interface interactions from the AtNTR_CC. One of the most important interactions for maintaining the distance of catalytic residues is hydrogen bond interaction between C153 and T430 in AtNTR_CC (Figure 7F). However, the shifted Trx domain in AtNTR_AC forms a different or weak interactions (Figure 7D and 7E). Hence, the different interaction and unfavorable linker binding in AtNTR_AC system induced a considerably long distance between TrxR C153 and disulfide bond of Trx C387–C390 and thus cause the inability of electron transfer reaction of the hybrid protein.

In the RMSF analysis for the two systems, the variable residues in PD domain and the linker region were found to have more dynamic motions in AtNTR_AC system, whereas the other domains had similar behavior with that of AtNTR_CC system (Figure 6A). The essential dynamics result was also showed that the most dynamic movement appeared in the linker region (Figure 8). Both RMSF and ED analyses suggest that the unstable linker region and shifted Trx domain in AtNTR_AC are major factor of the formation of farther distance affecting the inability of electron transfer reaction.

### Binding modes of the cofactors

Binding modes of the cofactors (FAD and NADP^+^) were also studied to know the influence of it in both the systems. Differences in binding mode of NADP^+^ and the subsequent conformational change in AtNTR_AC system have led to the observed differentiation compared with AtNTR_CC system. Though difference in binding of NADP^+^ persists between the two systems, no such major difference could be seen in FAD binding. Hence, it can be inferred that the cofactor NADP^+^ binding has significant effect on both conformation and function of the AtNTR_CC and AtNTR_AC systems. From our analyses, it was interesting to uncover a strong hydrogen bonding interaction between S167 and NADP^+^ in AtNTR_AC system, which was well maintained throughout the simulation from its initial binding mode. Such an interaction presumably might have led to the different orientation of NADP^+^ binding in AtNTR_AC system compared to AtNTR_CC system. This result suggests that, unfavorable linker binding may have influence on conformational changes of Trx domain with different binding mode of cofactor.

## Conclusions

In order to find out the reason of decreased electron transfer from TrxR to Trx domain in the hybrid protein AtNTRA-(Trx-D) even though the protein is forming similar structure with AtNTRC, reasonable molecular models of the enzymes AtNTRC & AtNTRA-(Trx-D) were constructed by homology modeling approaches and two MD simulations (AtNTR_CC and AtNTR_AC) on the proteins with same cofactors such as NADP^+^ and FAD. Both simulations were successfully performed and analyzed. Structural differences of entire and active site have been found from the superimposed representative structures of the two systems. From the active site comparison, we noticed that the distance between TrxR C153 and disulfide bond of Trx C387-C390 in active site of AtNTR_AC system is much farther than corresponding distance of AtNTR_CC during the simulation time. Due to the far distance between TrxR and Trx key cysteine residues in AtNTR_AC system, the system was not able to transfer electron from TrxR domain to Trx domain. The following reasons induced a considerably long distance between TrxR C153 and disulfide bond of Trx C387–C390; i) Unfavorable linker binding was relatively occurred in AtNTR_AC system when compared with stable AtNTR_CC system; ii) relative position of the Trx domain was changed by unstable linker region; iii) the different or weak interface interactions of Trx domain were observed. This study is one of the good examples for understanding the relationship between structure and activity in hybrid protein. In addition, this study can provide valuable insights upon understanding the electron transfer reaction in hybrid protein as well as AtNTRC.

## Materials and Methods

### Homology Modeling of AtNTRC and AtNTRA-(Trx-D)

To confirm the role of Trx domain in AtNTRA, the AtNTRA was connected with Trx domain from AtNTRC. The evolutionary relationship between these two enzymes were observed and categorized for preparing the structural model. The template structure (PDB ID: 1F6M) [15] which represents TrxR enzyme of *E. coli* was considered in the homology modeling of AtNTRC and AtNTRA-(Trx-D). The missing linker region was modeled using Protein Modeling tool from Discovery Studio 2.0. All the pairwise sequence alignment for comparison and modeling was carried out using the ClustalW sequence alignment program. The homology models of AtNTR_CC and AtNTR_AC together with the linker region and oxidized FAD and NADP^+^ were further refined using *MODELLER* [26], [27] module in Discovery Studio 2.0. The PROCHECK and ProSA-web programs were used to validate the stereochemical qualities of homology models [28], [29]. The binding position coordinates of oxidized FAD and NADP^+^ were obtained from the template in complex with cofactors.

### Molecular dynamics simulation

The MD simulations were run on the Linux multi-node parallel cluster computer. All the MD simulations were carried out using the GROMACS program (version 4.5.3) [30], [31] with Amber03 force field. The Gromacs topology files for the cofactors (Oxidized FAD and NADP^+^) were generated using the ACPYPE [32]. The initial structure was immersed in an orthorhombic water box and the net charge was neutralized by the addition of Cl^−^ and Na^+^ counterions. Long range electrostatics were handled using the particle mesh Ewald method [33]. In a system, protein alone consists of 13,720 atoms and the entire system is made up of approximately 142,000 atoms, which includes ∼42,500 water molecules (Table 1). The steepest descent energy minimization was used to remove possible bad contacts from the initial structures until energy convergence reached 2,000 kJ/(mol·nm). The systems were subject to equilibration at 300 K and normal pressure constant (1 bar) for 100 ps under the conditions of position restraints for heavy atoms and LINCS constraints [34] for all bonds. For the systems considered for study, production runs were performed under periodic boundary conditions with NPT ensemble. Cutoff distances for the calculation of the electrostatic and Lennard–Jones interaction were 0.9 and 1.4 nm, respectively. The time step of the simulation was set to 2 fs, and the coordinates were saved for analysis every 10 ps.

### Essential dynamics Analysis

The ED analysis method was applied to study the core or net protein motions from the huge amount of simple vibrational motions. The calculation was performed using trjconv, g_covar and g_anaeig modules of GROMACS. The ED procedure is equivalent to a multidimensional linear least squares fit of the trajectory, where the first eigenvector corresponds to the direction that fits best to the ensemble of configurations, the second to the second best, etc. The principle of such a multidimensional fitting was applied to protein dynamics for the first time by Garcia [35]. This analysis can filter out unessential motions (noise) and decomposes the overall motion into individual modes (directions of motions), which belong to individual eigenvectors with particular eigenvalues, derived by diagonalization of the covariation coordinate matrix from the atomistic MD trajectory [36], [37]. Because the ED analysis is mostly used on short 1–2 ns trajectories, it is useful to have an analysis of the statistical relevance and stability of the motions observed [38]. The motions in the essential space are often linked to the biological function of the protein.

## Supporting Information

Figure S1 **Ramachandran plots and z-scores obtained from Procheck and ProSA-web programs.** The 80.9%, 81.36%, and 86.1% residues of the template *E. coli* TrxR-Trx complex (A), AtNTR_CC (B), and AtNTR_AC (C) structures are shown in most favored regions, respectively. The proper z-score value of −10.22, −8.69, and −9.69 was also obtained from ProSA-web for the template, AtNTR_CC, and AtNTR_AC structures, respectively. As the result, the structures were found within a range of scores generally found for native proteins of similar size which are experimentally determined protein chains in current protein data bank. These validation results revealed that our homology models were well constructed.
(TIF)

Figure S2 **Superimposed structures obtained from additional MD simulation of AtNTR_AC without the linker region.** Superimposition of the representative structures between AtNTR_AC (red for subunit A and light brown for B) and AtNTR_AC without the linker (yellow) systems. The orientation obtained from this simulation is similar with that of AtNTR_CC.
(TIF)
