# Supplementary figures and images for: Comparative Molecular Modeling Study of Arabidopsis NADPH-Dependent Thioredoxin Reductase and Its Hybrid Protein
Source: PLoS One. 2012 Sep 27;7(9):e46279. doi: 10.1371/journal.pone.0046279 (PMC3459921; doi:10.1371/journal.pone.0046279)

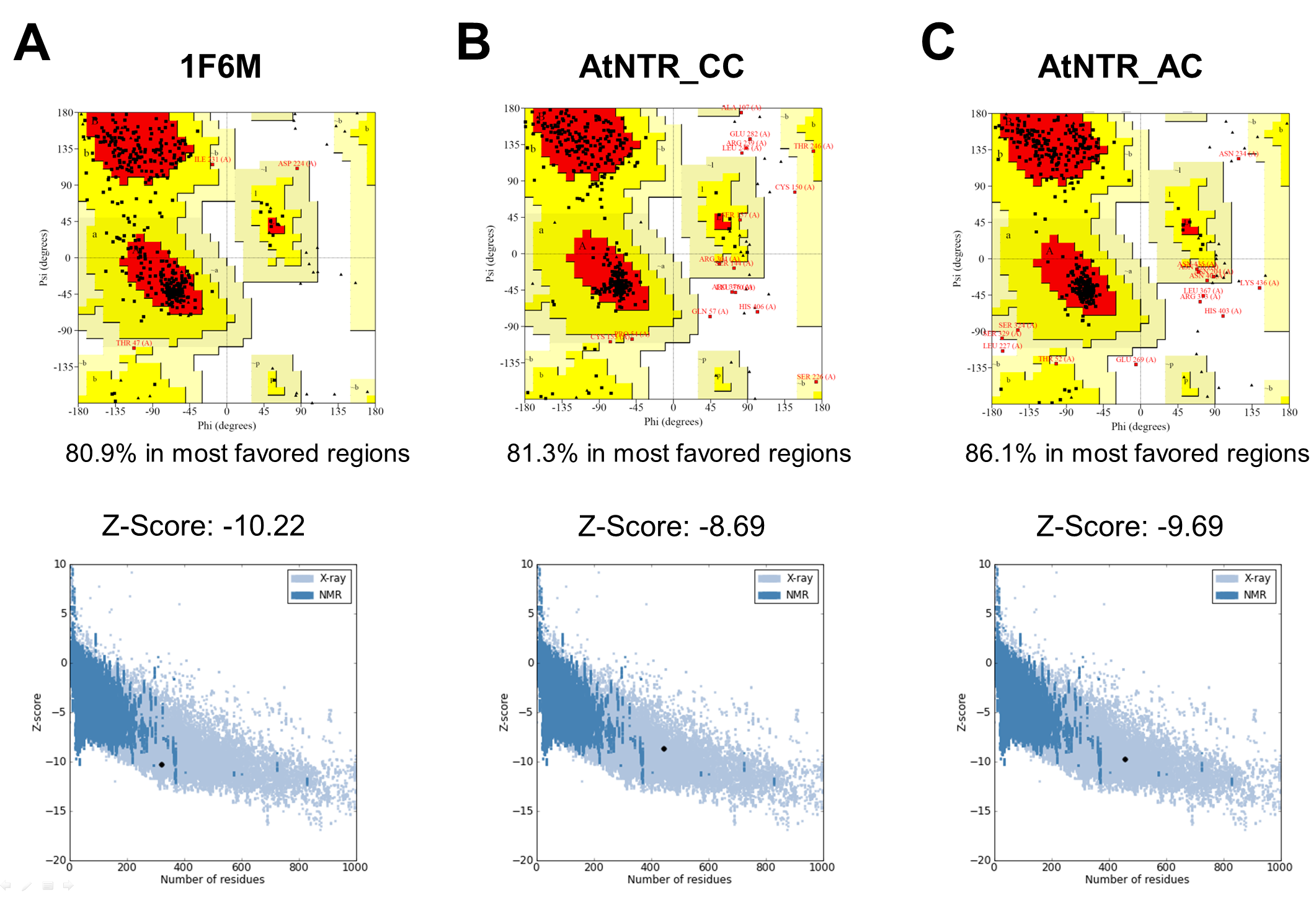

Supplement: Figure S1 — Ramachandran plots and z-scores obtained from Procheck and ProSA-web programs. The 80.9%, 81.36%, and 86.1% residues of the template E. coli TrxR-Trx complex (A), AtNTR_CC (B), and AtNTR_AC (C) structures are shown in most favored regions, respectively. The proper z-score value of −10.22, −8.69, and −9.69 was also obtained from ProSA-web for the template, AtNTR_CC, and AtNTR_AC structures, respectively. As the result, the structures were found within a range of scores generally found for native proteins of similar size which are experimentally determined protein chains in current protein data bank. These validation results revealed that our homology models were well constructed. (TIF) [file pone.0046279.s001.tif]

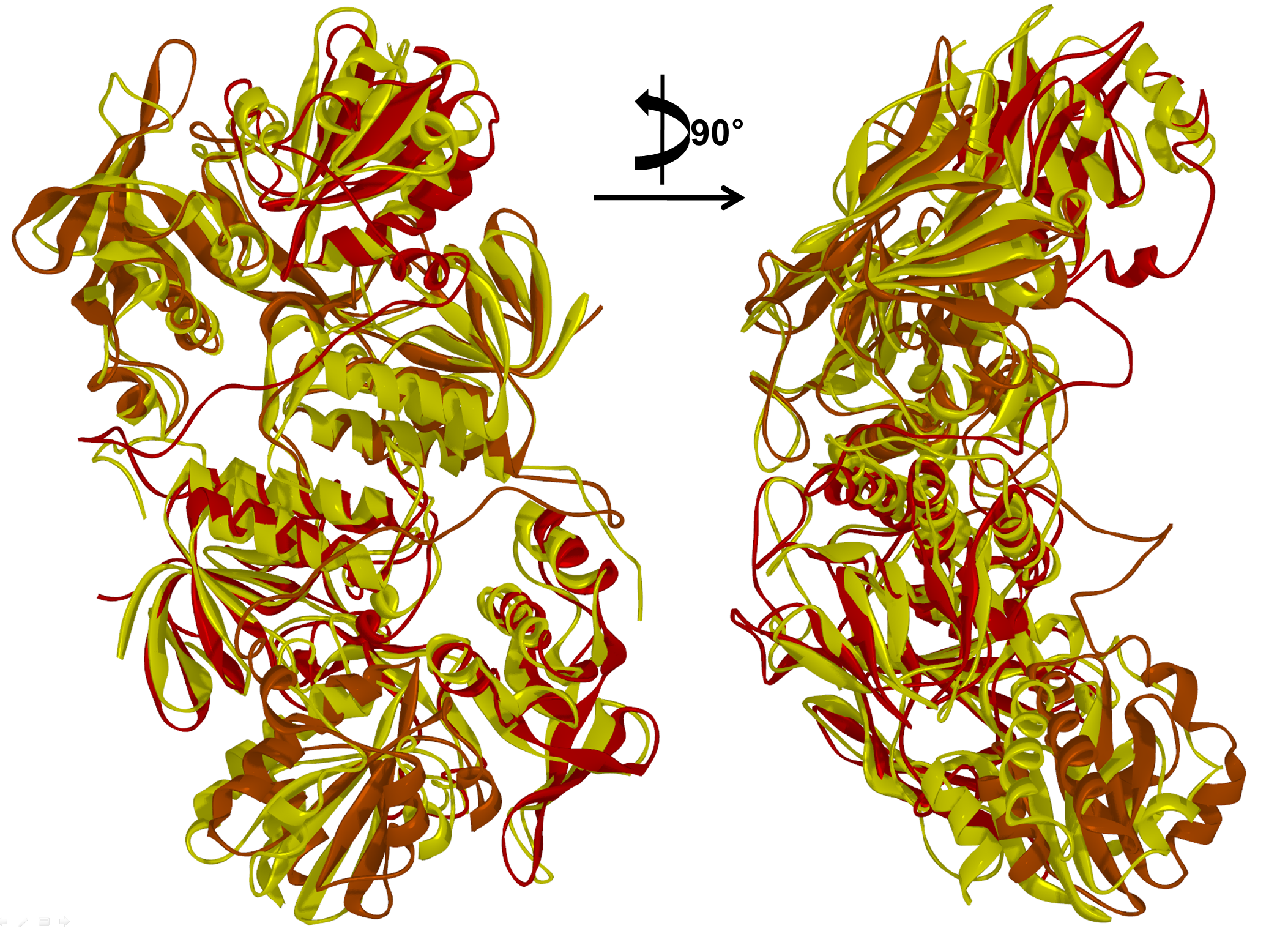

Supplement: Figure S2 — Superimposed structures obtained from additional MD simulation of AtNTR_AC without the linker region. Superimposition of the representative structures between AtNTR_AC (red for subunit A and light brown for B) and AtNTR_AC without the linker (yellow) systems. The orientation obtained from this simulation is similar with that of AtNTR_CC. (TIF) [file pone.0046279.s002.tif]
